# Stereotactic Adaptive Radiation Therapy for Borderline Resectable or Locally Advanced Pancreatic Cancer to Minimize Gastrointestinal Toxicity (ARTIA-Pancreas): Protocol for a Single-Arm Prospective Trial

**DOI:** 10.2196/84607

**Published:** 2026-02-23

**Authors:** Lauren E Henke, Hyun Kim, Eric Laugeman, Steven Kohlmyer, Claire McCann, Kate Pietrovito, Kenneth Russell, Jennifer Woo, Alex T Price

**Affiliations:** 1 Department of Radiation Oncology, Seidman Cancer Center, University Hospitals, 10900 Euclid Avenue, Cleveland, OH, 44106, United States, 1 216-844-3951; 2 Department of Radiation Oncology, Siteman Cancer Center, Washington University School of Medicine, St. Louis, MO, United States; 3 Varian Medical Systems, LLC, A Siemens Healthineers Company, Palo Alto, CA, United States

**Keywords:** adaptive radiation, radiation, pancreatic cancer, pancreas, dose escalation

## Abstract

**Background:**

Computed tomography (CT)–guided online stereotactic adaptive radiotherapy (CT-STAR) allows for ablative radiation doses to be delivered to selected patients with borderline resectable (BR) or locally advanced pancreatic cancer (LAPC) or unresectable pancreatic cancer. However, the use of CT-STAR to deliver an ablative dose to the pancreas while minimizing gastrointestinal (GI) side effects to reduce acute and late toxicity rates compared to historic controls has yet to be prospectively evaluated.

**Objective:**

The primary objective of this prospective, single-arm, multicenter phase 2 clinical trial (Adaptive Radiation Therapy Individualized Approach [ARTIA]–Pancreas) is to evaluate the rate of acute grade 3+ GI toxicity in patients with BR/LAPC treated with ablatively dosed CT-STAR compared to historical controls.

**Methods:**

Patients with histologically or cytologically confirmed BR, locally advanced, or medically inoperable pancreatic adenocarcinoma are eligible for participation. Consenting and eligible patients will be treated with CT-STAR, delivering 5 fractions over 1 to 2 weeks with daily adaptation based on anatomic changes observed with onboard cone beam CT imaging. The primary end point of this trial is the rate of acute patient-reported grade 3+ treatment-related GI toxicities, assessed at 90 days post CT-STAR and compared to a historical control rate of 20%. The key powered secondary end point is the rate of long-term patient-reported grade 3+ treatment-related GI toxicities, evaluated at 12 months post CT-STAR and compared to a historical control rate of 25%. Additional secondary end points include overall survival, local (in-field) control rates, and distant progression-free survival at 1 and 2 years post CT-STAR.

**Results:**

Study completion is anticipated in February 2029, and the final study results will be published upon completion of the study.

**Conclusions:**

ARTIA-Pancreas represents the first prospective phase 2 clinical trial to evaluate whether CT-STAR can reduce the rate of acute patient-reported GI toxicities in patients with BR/LAPC compared to historical controls. Findings from this clinical trial will provide evidence for safely and effectively incorporating ablatively dosed adaptive radiotherapy into treatment regimens for this population.

## Introduction

The only known curative intervention for patients with pancreatic cancer is surgical resection [1]. However, only approximately 20% of patients are clearly eligible for curative intent resection at the time of diagnosis, while 30% to 40% of patients present with either borderline resectable or locally advanced disease [2]. In patients with borderline resectable (BR) or locally advanced pancreatic cancer (LAPC), neoadjuvant chemotherapy and/or chemoradiotherapy are typically used for the purpose of tumor downstaging and affording the opportunity for curative resection [3]. Despite neoadjuvant treatment, only 20% to 30% of patients with BR/LAPC are typically converted to resectability, highlighting the need for improvements in these chemo- and radiotherapies to contribute to or provide definitive therapy [3]. Computed tomography (CT)–guided stereotactic adaptive radiotherapy (CT-STAR), a recent technological advancement in the radiotherapeutic space, may overcome the shortcomings of conventional radiotherapy to improve care for BR/LAPC.

Historically, conventional radiotherapy has shown only modest use in patients with pancreatic cancer, with randomized phase 3 clinical trials demonstrating a lack of survival benefit and only modest local control gains in patients treated with conventional chemoradiotherapy versus chemotherapy alone [4,5]. Dose-escalated conventional radiotherapy may convert 20% to 30% of unresectable patients to resectability [3] but imparts significant side effects, with rates of acute grade 3+ nonhematologic and gastrointestinal (GI) toxicity as high as 20% to 30% [3,4,6,7].

Stereotactic body radiotherapy (SBRT), which leverages fewer treatment days and higher dose conformality compared to conventional radiotherapy, has emerged as an alternative approach for achieving local disease control in patients with pancreatic cancer. However, ablative CT-guided pancreatic SBRT has been shown to induce similarly (>20%) concerning rates of grade 3+ GI toxicity [8] when target coverage is prioritized over organ-at-risk (OAR) protection. In patients with unresectable pancreatic cancer, studies suggest that reducing treatment margins and building in constrained planning-organ-at-risk volumes may partly mitigate acute GI toxicity following ablative SBRT [9-11]. Even so, these approaches do not consistently lead to meaningful reductions in late GI toxicity [12] and/or result in intentional limitation of tumor coverage. Therefore, historical use of ablative SBRT has been limited to near-palliative dosing regimens [13] or to use in patients with highly selective anatomic presentations [9,10,14].

Online adaptive radiotherapy (ART), a form of radiotherapy in which interfraction changes to the radiation dosimetry plan are made in response to observed changes in tumor and OAR anatomy, has emerged as a feasible and safe mechanism to deliver ablative SBRT to patients with abdominal cancer [15,16]. In patients with tumor locations with direct OAR abutment or invasion that would traditionally preclude treatment with ablative SBRT, online adaptive planning and delivery may enable safe delivery of ablative SBRT [17]. Findings from both a small, multi-institutional retrospective analysis and a recent phase 2 clinical trial suggest that dose escalation enabled by ART may improve 2-year overall survival while reducing grade 3+ GI toxicity in patients with BR/LAPC [18-20]. Furthermore, single-institution series and prospective analyses indicate that daily ART with magnetic resonance (MR) guidance may allow for safe incorporation of elective nodal irradiation (ENI) volumes with ablative doses [21,22], which is desirable per current treatment guidelines [23], but historically limited due to concerns of toxicity [24,25].

The delivery of ART is challenged by the need for high-quality daily on-board imaging, which is not achievable through conventional CT and has historically required MR-guided adaptive platforms. MR-guided adaptive platforms are costly and require specialized resources, restricting clinic access to ART from both financial and resource perspectives [26]. Recent technological improvements to conventional cone beam CT (CBCT) now permit CT guidance of ART using a ring-gantry linac (Ethos, Varian Medical Systems) [27]. Specifically, images acquired via an advanced onboard kV-CBCT technology have been found to approach the quality of diagnostic CT simulation images, allowing high-quality visualization and delineation of targets and OARs, providing a more accessible alternative to MR platforms [28].

Currently, there are limited data on the potential of CT-STAR to deliver ablative SBRT to the pancreas and ENI volumes. Washington University prospectively evaluated, in silico, the feasibility of using the kV-CBCT-based STAR system for delivery of ART to patients with unresectable pancreatic cancer [29]. CT-STAR was found to be feasible, with successful workflow and creation of online adaptive pancreas SBRT plans that met strict OAR constraints in 97.5% of fractions [29]. CT-STAR was also found to be dosimetrically advantageous, and subsequently, the first pilot study (N=8 participants) of CT-STAR for ablative treatment of pancreatic cancer was reported [30].

The aim of the Adaptive Radiation Therapy Individualized Approach (ARTIA)-Pancreas is to conduct a large (N=134 participants), prospective, single-arm, multicenter phase 2 clinical trial to evaluate the efficacy of CT-STAR in delivering ablative radiotherapy dose for BR/LAPC while reducing the rate of acute grade 3+ GI toxicity compared to historical controls (NCT05764720; *A Prospective Trial of Stereotactic Adaptive Radiation Therapy for Borderline Resectable/Locally Advanced Pancreatic Cancer: An Individualized Approach to Minimizing Gastrointestinal Toxicity, ARTIA-Pancreas*).

## Methods

### Study Objectives and Design Overview

The primary objective of ARTIA-Pancreas is to evaluate the rate of acute (90-d) grade 3+ GI toxicity resulting from ablatively dosed CT-STAR administered to patients with BR/LAPC compared to a historical rate of 20%. It is hypothesized that CT-STAR will reduce acute grade 3+ GI toxicity in this patient population, with an effect size of 10%.

ARTIA-Pancreas is also hierarchically powered to evaluate the key secondary objective of rate of late (12 mo) grade 3+ GI toxicity from ablatively dosed CT-STAR administered to patients with BR/LAPC compared to historical rates. We hypothesize that CT-STAR will reduce late grade 3+ GI toxicity, with a 15% effect size, from a 25% historical rate. Other secondary end points that will be evaluated include (1) overall survival at 12 and 24 months post completion of CT-STAR, (2) local (in-field) control rates at 12 and 24 months post completion of CT-STAR, and (3) distant progression-free survival at 12 and 24 months post completion of CT-STAR.

Exploratory objectives, along with associated end points, of ARTIA-Pancreas are listed in Table S1 in Multimedia Appendix 1.

**Figure 1.**
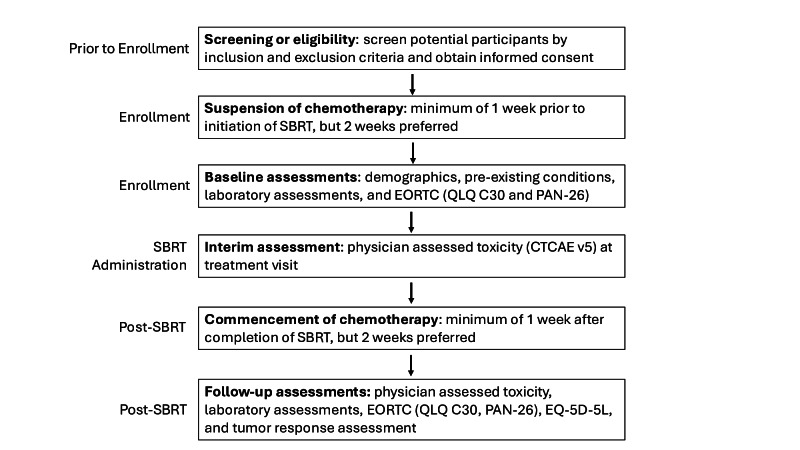
Adaptive Radiation Therapy Individualized Approach (ARTIA)–Pancreas study schema. CTCAE: Common Terminology Criteria for Adverse Events; EORTC: European Organization for Research and Treatment of Cancer; SBRT: stereotactic body radiotherapy; QLQ C30: Core Quality of Life Questionnaire; PAN-26: Pancreatic Cancer Module for Assessing Health Related Quality of Life.

The design of this prospective, single-arm phase 2 clinical trial is depicted in Figure 1. Briefly, potential participants are screened for eligibility according to inclusion and exclusion criteria (see “Participant Eligibility and Enrollment” section), and informed consent is obtained from eligible participants. Chemotherapy is suspended 1 to 2 weeks prior to CT-STAR treatment, and baseline assessments are conducted by the treatment team (Table 1). During the course of ablatively dosed CT-STAR administration (50 Gy in 5 fractions), physicians review the daily ART plans and perform interim assessments (Table 1) of GI toxicity.

One to two weeks after the completion of CT-STAR, chemotherapy may be reinitiated, and follow-up assessments are conducted in accordance with study end points (Table 1).

**Table 1.** Study assessment timeline and frequency.

Data required	0	Pre	RT^a^ 1‐2	Post 1 week	Post 6 week	Post 3 months	Post 6 months	Post 12 months	Post 18 months	Post 24 months
Consent	✓									
Patient demographics	✓									
PMH^b^	✓									
Volume imaging	✓					✓	✓	✓	✓	✓
RECIST^c^	✓					✓	✓	✓	✓	✓
CBC^d^	✓		✓	✓	✓	✓	✓	✓		
CMP^e^	✓		✓	✓	✓	✓	✓	✓		
CA^m^ 19‐9	✓		✓	✓	✓	✓	✓	✓		
Pregnancy	✓									
RT planning		✓								
CT-STAR^g^			✓							
MD review^h^			Each fx							
AE^i^ assessment			OTV^j^		✓	✓	✓	✓	✓	✓
EORTC^k^		✓			✓	✓	✓	✓		
EQ-5D-5L		✓			✓	✓	✓	✓		

a RT: radiation therapy.

b PMH: past medical history.

c RECIST: Response Evaluation Criteria for Solid Tumors.

d CBC: complete blood count.

e CMP: comprehensive metabolic panel.

f CA: carbohydrate antigen.

g CT-STAR: computed tomography–guided online stereotactic adaptive radiotherapy.

h MD review: real-time physician review of adapted plan.

i AE: adverse event.

j OTV: on treatment visit.

k EORTC: European Organization for Research and Treatment of Cancer.

### Site Credentialing and Quality Assurance

To maintain study rigor and quality across participating institutions, sites are credentialed prior to activation and required to upload planning images for the first two patients for central peer review. As a part of site credentialing, 2 benchmark cases (pancreas head and pancreatic tail tumor locations) consisting of a simulation CT and an Ethos CBCT are provided to the site, which will then prepare and submit the reference dosimetry plan as well as an adapted plan for approval by a centralized review committee for both clinical presentations.

After trial site activation, sites upload the simulation CT and pretreatment plan for the first 2 enrolled participants for central peer review 4 days prior to the planned start of external beam radiation therapy. Within 1 business day of completion of the participants’ first CT-STAR fraction, sites upload the deidentified daily fraction digital imaging and communications in medicine (DICOM)-RT data for clinical accuracy and protocol adherence review by the centralized committee. Central peer review is repeated every 5 subsequent participants or if a site has not enrolled a participant in 6 months.

### Participant Eligibility and Enrollment

Patients aged 18 years or older with histologically or cytologically confirmed BR, locally advanced, or medically inoperable (based on the National Comprehensive Cancer Network criteria) pancreatic adenocarcinoma are eligible for participation in this phase 2 clinical trial. Key exclusion criteria include a history of radiotherapy within the projected treatment field or prior, intercurrent, or planned treatment with an investigational agent related to pancreatic cancer diagnosis within 90 days of CT-STAR. Patients of all genders, races, and ethnic groups are eligible for ARTIA-Pancreas. Additional inclusion and exclusion criteria are listed in Textbox 1.

Textbox 1. **Additional participant inclusion and exclusion criteria.**

**Inclusion criteria**

- Eastern Cooperative Oncology Group performance status 0‐1.
- Receipt of at least 2 months of lead-in chemotherapy (regimen at the discretion of the treating physician) prior to planned initiation of computed tomography–guided online stereotactic adaptive radiotherapy (CT-STAR).
- Limited regional lymphadenopathy. Node+ patients are restricted to those with up to 3 clinically involved (≥1 cm on cross-sectional imaging or pathologically proven) nodes provided that the lymph nodes are adjacent to the primary tumor.
- Able to interrupt systemic therapy at least 1 week prior to planned start of CT-STAR (2 wk preferred) lasting for the duration of CT-STAR and continuing for at least 1 week following end of CT-STAR (2 wk preferred).
- Capable of single end-exhale breath-hold of at least 20 seconds in duration and of repeated end-exhale or deep inspiratory breath-hold of at least 10 seconds in duration upon verbal instruction.
- Anatomy of target and adjacent organs-at-risk adequately visualized on Ethos simulation imaging, as determined by treating and study physicians.
- Able to understand and willing to sign an institutional review board–approved written informed consent document (or that of legally authorized representative, if applicable).

**Exclusion criteria**

- Any additional cancer diagnosis made and/or treated within the preceding year (other than nonmelanoma skin cancer).
- Any history of prior malignancy (independent of time frame), with evidence of persistent or progressive disease (other than nonmelanoma skin cancer).
- Uncontrolled intercurrent illness, including but not limited to ongoing or active infection, symptomatic congestive heart failure, unstable angina pectoris, cardiac arrhythmia, or psychiatric illness or social situations that would limit compliance with study requirements.
- Pregnant and/or breastfeeding (patients of childbearing potential must have a negative pregnancy test within 14 d of study entry).
- Gross tumor invasion of the stomach or duodenum (defined either radiographically or endoscopically).

### Ethical Considerations

The protocol was approved by the University Hospitals Institutional Review Board (IRB) of the University Hospitals Cleveland Medical Center (protocol STUDY20240615). All participants are required to give written informed consent, indicated by a dated signature of the participant or legal representative, in compliance with International Organization for Standardization (ISO) 14155:2020 (WCG protocol 20225669). Personnel delegated by the principal investigator to conduct the informed consent process must have documented training in both good clinical practice and the study protocol. Patients are compensated ($1,000 - $1,300 USD) for trial participation, which is dependent on the site of enrollment and visit completion. Authorization to collect research data from participants’ medical records is obtained at the time of informed consent. Only deidentified data are entered into the electronic database where data are stored, and participants will be represented by a unique identifier in the database. All efforts are made to remove participant-identifying information and to deidentify data before entrance into the study database or transmitting the data to the sponsor. All reasonable efforts are made to protect the privacy of participants.

### Baseline Assessments

Baseline assessments include patient demographics, past medical history, complete blood count, pregnancy test, and comprehensive metabolic panel. In addition, physicians evaluate the patient’s oncologic status through carbohydrate antigen 19‐9 testing, Response Evaluation Criteria for Solid Tumors scoring, and volumetric imaging of tumors. Quality of life is also evaluated through the patient-reported European Organization for Research and Treatment of Cancer and EQ-5D-3L questionnaires.

### Pretreatment Anatomy Assessment and Radiotherapy Planning

To assess whether participants’ anatomy can be visualized adequately with CBCT adaptive treatment, all patients undergo both a CT simulation consisting of an end-exhale CT and a 4D CT, as well as an Ethos CBCT. Participants will be in the supine position, and all imaging extends from the superior aspect of the dome of the liver to the iliac crest. Participants with adequately visualized anatomy on CBCT then proceed to dosimetric treatment planning.

Investigators are provided with a contour atlas, which shows example contours for primary tumors in the head or neck and tail of the pancreas as well as for accompanying ENI. Target and normal structure contouring guidelines were developed to meet the clinical goals of ARTIA-Pancreas and are described for gross tumor volume_5000, clinical target volume_3300, planning target volume (PTV)_5000, and PTV_3300 as well as for the stomach, duodenum, small bowel, large bowel, spinal cord, left and right kidney, and liver.

Dose-volume histogram information for the target volumes and surrounding critical structures is mandatory. Minimum PTV coverage goals are 95% of the volumes to be covered by 25 Gy, with a reduction in PTV dose allowed in areas of OAR and target overlap. The initial treatment plan is created such that the plan will normalize to all priority 1 OAR (eg, stomach, duodenum, small and large bowel, spinal cord, and solitary kidney) objectives (Table 2). As such, OAR dose constraints function as strict constraints in treatment planning for both initial and adaptive (CT-STAR) plans, and gross tumor volume, PTV_5000, and PTV_3300 coverage is sacrificed, if necessary, to meet the OAR dose constraints.

**Table 2.** Organ-at-risk and target planning objectives, goals, and suggested planning weight priorities for the Ethos 2.0 treatment planning system optimization algorithm.

Name of structure	Suggested planning priority	Dosimetric parameter	Per protocol	Variation acceptable^a^
Stomach	1 4	V3300 cGy V3000 cGy	≤0.5 cc ≤5.0 cc	None^b^
Duodenum	1 4	V3300 cGy V3000 cGy	≤0.5 cc ≤5.0 cc	None
Small bowel	1	V3300 cGy	≤0.5 cc	None
Large bowel	1	V3300 cGy	≤0.5 cc	None
Spinal canal	1 3	V2500 cGy V1500 cGy	≤0.5 cc ≤0.5 cc	None None
Kidneys	3 and 1	Mean dose	≤1500 cGy	≤1800 cGy
Solitary kidney (if solitary kidney present)	— 1	V1000 cGy Mean dose	≤10% ≤1000 cGy	45% None
Kidney_L	3	Mean dose	≤1500 cGy	None
Kidney_R	3	Mean dose	≤1500 cGy	None
Uninvolved liver	2 R 1	Mean dose V2000 cGy Critical volume of 700 cc	≤1500 cGy ≤33% ≤1500 cGy	None None None

a Parameters not meeting the variation acceptable limits will lead to rescheduling of treatment for the following day.

b “None” means no acceptable variation permitted.

### Dose Prescription and Delivery Considerations

Radiotherapy treatment consists of SBRT delivered in 5 fractions once daily or once every other day in 1 to 2 weeks. Participants receive a planned dose of 50 Gy in 5 fractions to the PTV with 33 Gy in 5 fractions to the ENI volume, with strict OAR sparing of luminal GI OARs prioritized over target coverage.

An exhale breath-hold approach using ancillary, commercially available respiratory management systems is used for respiratory gating. At the time of treatment, an end-exhale breath-hold CBCT daily alignment image is acquired to establish a new reference threshold based on the daily anatomy. The participant’s respiratory motion is monitored using a respiratory trace generated and displayed to the treatment team for manually triggered breath-hold respiratory gating of radiation delivery. To monitor intrafraction participant motion, CBCT can be repeated a maximum of 10 times per fraction in the breath-hold position.

### Daily Treatment Workflow and Assessments

For daily adaptation of SBRT, participants are imaged using CBCT at an end-exhale breath-hold position. The ART team uses clinical discretion to determine if the image quality is appropriate for target alignment and OAR delineation. If on 3 consecutive attempts prior to delivery of the first fraction, the CBCT image quality is poor, precluding organ visualization and adaptive planning, then the participant is removed from the study and pursues institutional standard-of-care treatment. Once adaptive planning optimization is complete, the adapted plan is compared to the scheduled plan, and any adapted plan or scheduled plan that does not meet the SBRT dose constraints is not delivered. If the adaptive plan meets all objectives and the scheduled plan does not, the adaptive plan is selected for treatment. If both plans meet SBRT dose constraints, the adapted plan is selected over the scheduled plan only if the PTV coverage goals increase by more than 5%.

In addition to the review of the adaptive CT-STAR plan at each fraction, adverse events are evaluated during treatment visits. Specifically, grade 2+ adverse events that are attributed by the physician as possibly, probably, or definitely related to ART are recorded and classified per the Common Terminology Criteria for Adverse Events (CTCAE) v5.0.

### Follow-Up Assessments

Participants are assessed at 6 weeks and at 3, 6, 12, 18, and 24 months after completion of CT-STAR treatment. CTCAE v5.0-evaluated adverse events are reported at all of these time points, while patient-reported quality of life is ascertained via the European Organization for Research and Treatment of Cancer and EQ-5D-3L at 6 weeks and 3-, 6-, and 12-months after CT-STAR. Volumetric imaging and tumor response, both having occurred at baseline, are repeated at 3, 6, 12, 18, and 24 months posttreatment to determine the patients’ response to radiotherapy.

### Study End Points

The primary end point for ARTIA-Pancreas is the rate of acute (90 d) grade 3+ treatment-related GI toxicities as defined by the CTCAE v5.0 and compared to the historical rate of 20%. The trial’s key powered secondary end point is the rate of late (12-month) grade 3 or higher treatment-related GI toxicities as defined by the CTCAE v5.0 criteria and compared to the historical rate of 25%. Additional secondary end points include (1) a Kaplan-Meier estimate of the rates of overall survival at 12 and 24 months post completion of CT-STAR; (2) local (in-field) control rates at 12 and 24 months post completion of CT-STAR, defined as stable disease, partial response, or complete response by the Response Evaluation Criteria in Solid Tumors criteria; and (3) the Kaplan-Meier estimates of distant progression-free survival at 12 and 24 months post completion of CT-STAR.

Exploratory end points of ARTIA-Pancreas are listed in Table S1 in Multimedia Appendix 1.

### Statistical Analysis

#### Primary End Point

ARTIA-Pancreas is powered to the primary end point. It is hypothesized that CT-STAR will reduce the rate of CTCAE v5.0 grade 3+ GI toxicity compared to non-ART, where the historical acute toxicity rate is 20% or higher. It is hypothesized that using CT-STAR, the acute toxicity rate will be <10% for an effect size of 10% or more. A total of 107 evaluable participants (ie, participants with nonmissing data for the primary end point) are targeted. There must be ≤13/107 (12.1%; 95% CI 7.2%‐19.7%) participants with acute grade 3+ GI toxicity in the study. With this rule, the study’s 1-sided false positive error rate (α) is 0.023, and the power is 0.82. Given the possibility that participants could become eligible for surgical resection at the treated site following radiation, which would preclude toxicity assessment in those participants, we estimate a 20% attrition rate, and 134 participants will be targeted for enrollment.

CTCAE v5.0 grade 3+ GI toxicity compared to a historical rate of 20% will be analyzed by constructing a Wilson score 95% CI for the observed acute grade 3+ GI toxicity incidence rate. The CI’s upper limit being <20% will indicate rejection of the historical rate of 20% at a 1-sided *P*<.025 significance level (ie, success for the study’s primary end point).

#### Key Secondary End Point

ARTIA-Pancreas is also powered to analyze the key secondary end point, contingent upon a positive result for the primary end point. For the key secondary end point, it is hypothesized that CT-STAR will reduce late GI toxicity from a historical rate of 25% or greater to less than 10%. Given an evaluable 107 participants, there must be 17 (15.9%) participants or fewer with late grade 3+ GI toxicity in the study to reject a 25% or higher CT-STAR toxicity. With this rule, the key secondary end point’s 1-sided false-positive error rate is 0.016 when the true late toxicity rate is 25%, and the power is 0.98 when the true late toxicity rate is 10%.

If the key secondary end point is indeed evaluated, a Wilson score 95% CI will be constructed for the study’s observed 12-month grade 3+ GI toxicity incidence rate. The CI’s upper limit being less than 25% will indicate rejection of the historical rate of 25% at a 1-sided *P* value less than .025 significance level (ie, success for the study’s key secondary end point).

#### Secondary End Points

Twelve- and 24-month overall survival as well as distant progression-free survival will be evaluated by Kaplan-Meier analysis. All other secondary end points will be descriptively summarized.

#### Stopping Criteria

The sequential probability ratio test is being used to guard against an unacceptable toxicity rate during the study.

An expected toxicity rate of 10% and an unacceptable toxicity rate of 20% will be used with the total sample size of 134 participants. The probability of stopping early under the expected toxicity rate of 10% is set to .025, and the probability of stopping early when the true toxicity rate is 20% is set to .80.

### Data Collection, Management, and Monitoring

Data collection includes obtaining data in the form of case report forms (electronic and/or paper), patient-reported outcomes (electronic and/or paper), DICOM images from CT simulation, DICOM-RT data from the pretreatment, daily fraction DICOM and DICOM-RT data, investigator feedback (email and/or written), observations from sponsor representatives (email or written), responses to data queries, and copies of pertinent records (ie, participant charts and laboratory data). All data are recorded on case report forms that are supported by source documentation. While no imputation methods will be used for missing outcomes, sensitivity analyses will be conducted evaluating results for different assumptions of toxicity levels among participants who are withdrawn from the study.

To monitor safety and efficacy signals in real time, study staff are required to report serious adverse events to the IRB according to IRB reporting requirements and to the study sponsor within 72 hours of becoming aware of the event. Likewise, radiotherapy device issues that occur during study performance and protocol deviations are documented and reported to the IRB and sponsor per reporting requirements. Per the stopping criteria defined above, the study can be suspended for review by the sponsor and lead investigators if unacceptable toxicity is suspected.

## Results

The ARTIA-Pancreas trial opened for enrollment in May 2023. Primary completion is anticipated in February 2026, and study completion is anticipated in February 2029. The final study results will be published upon completion of the study.

## Discussion

### Overview

ARTIA-Pancreas is the first multicenter phase 2 clinical trial to evaluate the use of CT-STAR to minimize acute GI toxicity and result in favorable outcomes in patients with BR/LAPC. The study’s primary objective is to evaluate the rate of high-grade GI toxicity at 90 days following a 5-fraction course of CT-STAR, with the hypothesis that adaptive SBRT will reduce acute grade 3+ toxicity from the historical control rate of 20% to 10%. Importantly, ARTIA-Pancreas has been strategically designed to hierarchically power the evaluation of the key secondary end point, 12-month treatment-related CTCAE v5.0 grade 3+ GI toxicity incidence rate, compared to a historical control rate of 25%.

### Anticipated Results

Enrollment for ARTIA-Pancreas began in May 2023 and is anticipated to yield clinical findings shortly after completion in February 2028. The trial will provide evidence as to whether ablatively dosed radiotherapy that is adapted to the daily anatomy of each patient with BR/LAPC will reduce GI side effects. If, as is hypothesized, CT-STAR can reduce rates of treatment-related GI toxicity in this population, it may provide a rationale for the safe and effective incorporation of ablatively dosed CT-guided ART as a standard-of-care therapy.

### Limitations

Historically, delivery of ablatively dosed SBRT has been difficult to safely achieve in the upper abdomen due to the proximity and sensitivity of adjacent OARs as well as inter- and intrafraction organ motion. The ARTIA-Pancreas trial seeks to overcome this limitation by treating patients with BR/LAPC using an adaptive SBRT strategy that enables safe dose escalation by reshaping the target dose in response to changes in OAR proximity and movement.

ARTIA-Pancreas has the same limitations as any trial using historical comparators, including potential time confounding related to changes in clinical practice or affected populations. Evidence generated by ARTIA-Pancreas may lay the foundation for potential future randomized trials directly comparing the safety and efficacy of CT-guided ART platforms to MR-guided ART platforms and standard-of-care in patients with BR/LAPC.

Should ARTIA-Pancreas yield positive findings concerning the use of CT-STAR in patients with BR/LAPC, there are several noteworthy considerations for generalization of these data. First, findings from ARTIA-Pancreas may not be generalizable to all patients with locally advanced pancreatic cancer due to the study’s exclusion of participants with defined comorbidities as well as more advanced regional adenopathy. Second, despite being less resource-intensive than MR-guided ART, CT-guided ART still requires significant infrastructural, personnel, and financial commitments to successfully implement. While daily changes to patients’ radiotherapy plans may allow for reduced treatment-related side effects, they often require additional staffing and resource utilization [31]. To address these barriers to CT-STAR implementation, logistical and workflow learnings from large-scale, multicenter trials such as ARTIA-Pancreas can be disseminated across the clinical community.

The success of ARTIA-Pancreas depends on the adequate imaging of tumor targets and OARs, which could be affected by variability in imaging quality across centers. Elements of the study that address this include assessment of patient anatomy visualization on CBCT for the determination of final eligibility, site credentialing, and central quality assurance reviews of participant imaging and ART planning at scheduled intervals.

### Comparison With Prior Work

The historical framework for the use of radiotherapy in BR/LAPC is summarized in the “Introduction” section. The current clinical paradigm favors the use of ablative doses of SBRT with a strategy focused on minimizing adverse effects to adjacent normal tissues using daily adaptive techniques. In the recent phase I and retrospective studies, MR-based ART has been reported to improve the therapeutic index of SBRT for BR/LAPC, with encouraging local control rates of 70% to 80% at 2 years following treatment and a favorable toxicity profile [32]. While MR provides high-quality radiotherapy planning images, MR-guidance platforms require special construction, zoning, training, and staff presence that may impede widespread access to ART. In addition, patients may have MR imaging–incompatible medical devices or surgical implants that adversely impact the ability to deliver MR-guided ART.

ARTIA-Pancreas evaluates CT-, rather than MR-, guided ART [21,22] in an appropriately powered prospective clinical trial. The commercially available, advanced kV-CBCT imaging technology used in ARTIA-Pancreas can perform large field-of-view scans in the time of a single breath-hold and has demonstrated clinically acceptable target delineation and OAR daily anatomy visualization. Should the primary hypothesis of ARTIA-Pancreas be met, CT-guided ART may become a solution that achieves both clinical performance and potentially more widespread availability.

### Conclusions

The results of ARTIA-Pancreas will provide clinical evidence to support health care professionals in decision-making regarding the use of daily adaptive CT-STAR in the treatment of BR/LAPC.

## Supplementary material

10.2196/84607 Multimedia Appendix 1 Adaptive Radiation Therapy Individualized Approach (ARTIA)–Pancreas exploratory objectives and end points.

## Abbreviations

ART: adaptive radiotherapy
ARTIA: Adaptive Radiation Therapy Individualized Approach
BR: borderline resectable
CBCT: cone beam computed tomography
CT: computed tomography
CT-STAR: computed tomography–guided online stereotactic adaptive radiotherapy
CTCAE: Common Terminology Criteria for Adverse Events
ENI: elective nodal irradiation
GI: gastrointestinal
IRB: institutional review board
ISO: International Organization for Standardization
LAPC: locally advanced pancreatic cancer
MR: magnetic resonance
OAR: organ-at-risk
PTV: planning target volume
SBRT: stereotactic body radiotherapy
