# Supplementary material for: Stereotactic Adaptive Radiation Therapy for Borderline Resectable or Locally Advanced Pancreatic Cancer to Minimize Gastrointestinal Toxicity (ARTIA-Pancreas): Protocol for a Single-Arm Prospective Trial
Source: JMIR Res Protoc. 2026 Feb 23;15:e84607. doi: 10.2196/84607 (PMC12928687; doi:10.2196/84607)
Supplement: Multimedia Appendix 1 [file resprot-v15-e84607-s001.docx]

| **Table S1.** ARTIA-Pancreas exploratory objectives and endpoints | |
| --- | --- |
| **Objective(s)** | **Endpoint(s)** |
| Determine the incidence of late Grade 2 and Grade 3+ toxicity rates at 1- and 2-years after completion of CT-STAR | Incidence of CTCAE v5.0 late Grade 2 and Grade 3+ toxicity rates 1- and 2-years after completion of CT-STAR |
| Evaluate Ca 19-9 kinetics before and after CT-STAR | Quantitative evaluation of Ca 19-9 level at pre-treatment baseline, fraction 1, 1-week post-CT-STAR, 6-weeks post-CT-STAR, 3 months post-CT-STAR, and 6 months post-CT-STAR |
| Patient reported QoL outcomes pre- and post-CT-STAR utilizing standard validated questionnaires. | EORTC QLQ C30- and EORTC –PAN-26-demonstrated patient-reported QoL scores at baseline, 3 months, and 6 months after completion of CT-STAR |
| Compliance with target and OAR contour guidelines as well as on-table adaptive treatment planning objectives | Qualitative and quantitative analysis of compliance with target and OAR contour guidelines as well as on-table adaptive treatment planning objectives, as determined by retrospective, central review |
| Measure geometric agreement between breath-hold positions as determined by the respiratory motion management system vs. concomitant breath-hold CBCT image sets obtained before, during, and after CT-STAR delivery | Geometric agreement between breath-hold positions as determined by the on-board respiratory motion management system vs. concomitant breath-hold CBCT image sets obtained before, during, and after CT-STAR |
| Identify patient anatomic factors (e.g. air or metal artifact, obesity, prior abdominal surgeries, ascites) causing study withdrawal for inability to deliver treatment with CT-STAR | Qualitative and quantitative description of patient anatomic factors that were associated with study withdrawal for inability to delivery treatment with CT-STAR |
| Evaluation of online adaptive plan dosimetric predictors of acute and late toxicities as well as local tumor control | Quantitative correlation of daily online adaptive plan dosimetric indices and observed instances of acute and late toxicities as well as local tumor control |
| CT-STAR, CT-guided stereotactic adaptive radiotherapy; CTCAE, Common Terminology Criteria for Adverse Events; QoL, quality of life; OAR, organ-at-risk; EORTC, European Organization for Research and Treatment of Cancer; CBCT, cone beam computed tomography | |
